# Repeated measures of implementation variables

**DOI:** 10.3389/frhs.2023.1085859

**Published:** 2023-03-07

**Authors:** Dean L. Fixsen, Melissa K. Van Dyke, Karen A. Blase

**Affiliations:** Active Implementation Research Network, Inc., Chapel Hill, NC, United States

**Keywords:** implementation, scaling, measurement, validity, replication

## Abstract

It is commonly acknowledged that implementation work is long-term and contextual in nature and often takes years to accomplish. Repeated measures are needed to study the trajectory of implementation variables over time. To be useful in typical practice settings, measures that are relevant, sensitive, consequential, and practical are needed to inform planning and action. If implementation independent variables and implementation dependent variables are to contribute to a science of implementation, then measures that meet these criteria must be established. This exploratory review was undertaken to “see what is being done” to evaluate implementation variables and processes repeatedly in situations where achieving outcomes was the goal (i.e., more likely to be consequential). No judgement was made about the adequacy of the measure (e.g., psychometric properties) in the review. The search process resulted in 32 articles that met the criteria for a repeated measure of an implementation variable. 23 different implementation variables were the subject of repeated measures. The broad spectrum of implementation variables identified in the review included innovation fidelity, sustainability, organization change, and scaling along with training, implementation teams, and implementation fidelity. Given the long-term complexities involved in providing implementation supports to achieve the full and effective use of innovations, repeated measurements of relevant variables are needed to promote a more complete understanding of implementation processes and outcomes. Longitudinal studies employing repeated measures that are relevant, sensitive, consequential, and practical should become common if the complexities involved in implementation are to be understood.

## Introduction

Measurement of implementation variables in practice has been a challenge because of the complexities in human service environments, the novelties encountered in different domains (e.g., education, child welfare, global public health, pharmacy), and the ongoing development of implementation as a profession and as a science.

Greenhalgh et al. (1) conducted an extensive review of the diffusion and dissemination literature. They reflected a commonly held view when they concluded: “Context and “confounders” lie at the very heart of the diffusion, dissemination, and implementation of complex innovations. They are not extraneous to the object of study; they are an integral part of it. The multiple (and often unpredictable) interactions that arise in particular contexts and settings are precisely what determine the success or failure of a dissemination initiative.” For a science of implementation to develop, measures of implementation-specific independent and dependent variables must be established and used in multiple studies. Those variables and measures must be usable across the “multiple (and often unpredictable)” situations Greenhalgh et al. described.

Implementation is viewed by many as a process that takes time and planned activities at multiple levels so that innovations can be used fully and effectively and at scale (2). Yet, studies labeled as “implementation science” predominately use unique measures and one-time assessments of something of interest to the investigator (3, 4). Currently, avid readers of the “implementation science” literature are hard pressed to find a measure of an implementation-specific independent or dependent variable. Even when one is found, one data point at one point in time is a poor fit with the complexity of implementation as described in the literature. For example, Allen et al. (4) reviewed the literature related to the “inner setting” of organizations as defined by the Consolidated Framework for Implementation Research (CFIR). Consistent with previous findings from a review and synthesis of the implementation evaluation literature (3), Allen et al. found only one measure that was used in more than one study and noted that the definitions of constructs with the same name varied widely across the measures.

Repeated measures of multiple variables are needed to match the complexity of the practice, organization, and system environments in which changes must occur to support the full and effective uses of innovations in practice. Researchers have documented the years typically required to accomplish implementation goals (5, 6) even when skilled implementation teams are available (7). To advance a science of implementation, repeated measures and methods must be well suited to cope with research in applied settings where there are too few cases, too many variables, and too little control over multi-level variables that may impact outcomes (8, 9).

Implementation specialists and researchers who are doing the work of implementation and studying the results over time continually deal with complexity and confounders to accomplish their implementation practice and science aims (10). In their description of implementation practice and science, Fixsen et al. (10, chapter 16) proposed criteria for “action evaluation” measures that can be used to inform action planning and monitor progress toward full and effective use of innovations. Action evaluation measures are: (1) *relevant* and include items that are indicators of key leverage points for improving practices, organization routines, and system functioning, (2) *sensitive* to changes in capacity to perform with scores that increase as capacity is developed and decrease when setbacks occur, (3) *consequential* in that the items are important to the respondents and users and scores inform prompt action planning; repeated assessments each year monitor progress of action planning as capacity develops and outcomes are produced, and (4) *practical* with modest time required to learn how to administer assessments with fidelity to the protocol, and modest time required of staff to respond to rate the items or prepare for an observation visit.

While the lack of assessment of psychometric properties has been cited as a deficiency (11–13), what is missing from nearly all of the existing implementation-related measures is a test of consequential validity (14). That is, evidence that the variable under study, and the information generated by the measure of the variable, is highly related to using an innovation with fidelity and related to producing intended outcomes to benefit a population of recipients. Given that implementation practice and science are mission-driven (15), consequential validity is an essential test of any measure, an approach that favors external validity over internal validity (16, 17).

Galea (18), working in a health context, stated the problem and the solution clearly:

A consequentialist approach is centrally concerned with maximizing desired outcomes, and a consequentialist epidemiology would be centrally concerned with improving health outcomes. We would be much more concerned with maximizing the good that can be achieved by our studies and by our approaches than we are by our approaches themselves. A consequentialist epidemiology inducts new trainees not around canonical learning but rather around our goals. Our purpose would be defined around health optimization and disease reduction, with our methods as tools, convenient only insofar as they help us get there. Therefore, our papers would emphasize our outcomes with the intention of identifying how we may improve them.

By thinking of “our methods as tools, convenient only insofar as they help us get there” psychometric properties may be the last concern, not the first (and too often, only) question to be answered. The consequential validity question is “so what?” Once there is a measure of a variable it is incumbent on the researcher (the measure developer) to provide data that demonstrates how knowing that information “helps us get there.” Once a measure of a variable has demonstrated consequential validity then it is worth investing in establishing its psychometric properties to fine tune the measure. It is worth it because the variable matters and the measure detects its presence and strength.

This exploratory review was undertaken to “see what is being done” to measure implementation variables and processes in situations where achieving outcomes was the goal (i.e., more likely to be consequential). The interest is in measures that are relevant, sensitive, consequential, and practical. In particular, given the long-term and contextual nature of implementation work that often takes years to accomplish, the search is for studies that have used repeated measures to study the trajectory of implementation variables over time. For this review, a measure that has been used more than once in a study is an indication that the measure is relevant to the variable under study, sensitive to change in the variable from one data point to the next, consequential with respect to informing planning for change, and practical by virtue of being able to be used two or more times to study a variable.

## Materials and methods

The review was conducted within the Active Implementation Research Network (AIRN) EndNotes data base. The AIRN EndNotes data base contains 3,950 references (March 20, 2021) that pertain to implementation with a bias toward implementation evaluation and quantitative data articles. The oldest reference relates to detecting and evaluating the core components of independent variables (19). The most recent article describes over 10 years of work to scale up innovations in a large state system (20).

In 2003 the AIRN EndNotes data base was initiated by entering citations from the boxes of paper copies of articles accumulated by the authors since 1971, the year of the first implementation failure experienced by the first author (21). Beginning in 2003 AIRN EndNotes was expanded with references produced from literature searches conducted through university library services (3). Since 2006, articles routinely have been added through Google Scholar searches. Weekly lists of articles identified with the implementation-related search terms are scanned and relevant citations, abstracts (when available), and PDFs (when available) are downloaded into AIRN EndNotes. For inclusion in the database, articles reporting quantitative data are favored over qualitative studies or opinion pieces. Reflecting the universal relevance of implementation factors, the data base includes a wide variety of articles from multiple fields and many points of view. About 2/3 of the articles in AIRN EndNotes were published in 2000–2021.

The majority of articles in AIRN EndNotes published since 2000 include the Abstract and/or a PDF, and the full text of about half of all the articles has been reviewed by the authors and their colleagues. The reviewer of an article enters information in the “Notes” section of EndNotes regarding concepts and terms that relate to the evidence-based Active Implementation Frameworks as they are defined, operationalized, evaluated, and revised (3, 7, 15, 22–27). Given the lack of clarity in the implementation field, the Notes provide common concepts and common language that facilitate searches of the AIRN EndNotes data base.

For this study, the AIRN EndNotes data base was searched for articles that used repeated measures of one or more implementation variables. Using the search function in EndNotes, “Any Field” (i.e., title, abstract, keywords, notes) in the data base was searched using the word “measure” *and* the term “repeated,” or “longitudinal,” or “pattern,” or “stepped wedge.” The search returned 260 references. Because searches of the literature were less systematic in the pre-internet days, references published prior to the year 2000 were eliminated leaving 213 references. The title and abstract of each of the 213 articles was reviewed and those with apparent repeated measures of an implementation variable were retained (*n* = 58).

The full text of the remaining 58 references was reviewed. For the full text review, “repeated” was defined as two or more uses and “measure” was defined as the same method (observation, record review, survey, systematic interview) with the same content used at Time 1, Time 2, etc. No judgement was made about the adequacy of the measure or time frames. Thus, psychometric properties of a measure were not considered in the review. “Implementation” was defined as any specific support (e.g., training, coaching, leadership, organization changes) for the full and effective use of an identified innovation.

The full text review eliminated 26 articles. The reasons for elimination are provided in Table 1. For example, 13 articles were eliminated because the repeated measure concerned an intervention and not an implementation variable and 7 were eliminated because the same measure was not repeated from one time period to the next.

**Table 1:** Articles eliminated after full text review.

Reason for Eliminating an Article	Number of Articles
Intervention variables only - not a study of implementation	13
Same measure was not repeated	7
Measure development with a convenience sample	2
An open-ended interview - not a measure	1
Qualitative study - no measures	1
Reprint of an evaluation already included for study	1
Implementation variables identified but not measured	1

## Results

The search process resulted in 32 articles that met the criteria for a “repeated” “measure” of “implementation” variables: in 17 articles 2 or more variables were measured and in 15 articles one variable was measured. Fourteen (14) of the articles were published in 2000–2010 and 18 were published in 2011–2019.

As noted in Table 2, 23 different implementation variables were the subject of repeated measures. In Table 2 the Implementation Variable names are grouped using the Active Implementation Frameworks as a guide (10). The broad spectrum of implementation variables included innovation fidelity (assessed in 17 articles), sustainability (assessed in 8 articles), organization change (assessed in 6 articles), and scaling (assessed in 5 articles). Training, implementation teams, and implementation fidelity were the subject of 2 articles each.

**Table 2:** Implementation variables measured two or more times in the 32 articles.

Implementation Variable	Number of Articles
**Related to Implementation Stages**	
Exploration Stage	1
Community readiness	1
Organization readiness	1
Initial Implementation Stage	1
**Related to Implementation Drivers**	
Competency Drivers	1
Training	2
Coaching	1
Innovation fidelity	17
Organization Drivers	1
Organization change	6
Organization capacity	1
Leadership Drivers	1
**Other**	
Implementation Teams	2
Improvement cycles	1
Implementation capacity	1
Implementation fidelity	2
System change	1
Scaling	5
Sustainability	8
TA Collaboration	1
Attitude toward EBPs	1
Organization culture	1
Organization climate	1

Table 3 details the individual articles, the assessments they reported, and the implementation variables that were studied.

**Table 3:** The information in the table was sorted alphabetically based on the implementation Variable column (information regarding the implementation Variable can be found at www.activeimplementation.org).

Article	Repeated Measure	Implementation Variable
Strand et al. (28)	Each of the 6 sites selected an implementation team to carry out the initiative. Measures developed for this project included a key informant interview to assist in agency selection and an Organizational Readiness Assessment (ORA). The ORA was used across sites every 6 months. The ORA eight factors included three that aligned with the Organization driver, two factors that aligned with the Competency driver, two that aligned with the Leadership driver, and one stand-alone factor, Attitude Toward Evidence-based Treatment that consisted of one item.	Competency Drivers; Organization Drivers, Leadership Drivers; Attitude toward EBPs
Panzano et al. (29)	A longitudinal study designed to collect a range of interview, survey, and implementation outcome data in 91 agencies and relate the data from earlier stages to later stages. At 9-month intervals Panzano and colleagues followed a group of 91 agencies that had committed to and were funded to use one of several evidence-based programs in a state mental health system. The 91 agencies engaged in Exploration and Installation activities but 44 (48%) never used a selected program (i.e., did not reach the Initial Implementation Stage).	Exploration Stage; Initial Implementation Stage; Sustainability
Vernez et al. (30)	Assessed School staff commitment, Professional development, Adequacy of resources, External assistance, Internal facilitator, Feedback on instruction, and District support at 3 points: Yrs. 1–3, 4–6, 7 + using standard RAND-University of Washington principal and teacher surveys.	Implementation fidelity
Fixsen and Blase (21)	Implementation fidelity and organization sustainability measured repeatedly for 59 attempted replications of organizations using an evidence-based program with fidelity	Implementation fidelity; Sustainability
Fixsen et al. (31)	Assess implementation capacity development every six months for five years in state education systems. Measures assessed Leadership Investment (implementation roles and functions, coordination for implementation, leadership), System Alignment (implementation guidance documents, state design team), Commitment to Regional Implementation Capacity Development (resources for regional implementation capacity development, support for RIT functioning)	Implementation Teams; Scaling
Datta et al. (32)	The team set an aim of eliminating severe hypothermia and reducing moderate hypothermia by 50%. 41 data points over two years. Repeated PDSA Cycles with interventions to solve each set of problems exposed in the last cycle. Evaluated staff training (improving hand hygiene), administrative changes (team met weekly; supervised change; consistent supply of warm linen), facility changes (placement of charging leads for transportation incubators).	Improvement cycles; Organization change; Practitioner training
Lee and Cameron (33)	The Dual Diagnosis Capability in Addiction Treatment (DDCAT) index is a fidelity instrument for measuring alcohol and other drug treatment services’ capacity to provide comorbidity service to clients. Measures were taken the week prior to the PsyCheck training and 6 months after the training at each site (13 sites).	Innovation fidelity
Hardeman et al. (34)	A measure of fidelity was developed and tested across practitioners. Repeated measures of four sessions.	Innovation fidelity
McIntosh et al. (35)	Assessed fidelity annually for 5,331 schools over a 5-year course of implementing school-wide positive behavioral interventions and supports (SWPBIS). Four patterns of developing and sustaining fidelity were found.	Innovation fidelity; Sustainability
Tiruneh et al. (36)	Before and after data from 134 intervention health centers were collected in April 2013 and July 2015. [27 mos.] A BEmONC implementation strength index was constructed from seven input and five process indicators measured through observation, record review, and provider interview. The BEmONC implementation strength index score ranged between zero and ten. Assessments were made pre-post tailored support (including BEmONC training to providers, mentoring and monitoring through supportive supervision, provision of equipment and supplies, strengthening referral linkages, and improving infection-prevention practice) provided in a package of interventions to 134 health centers, covering 91 rural districts of Ethiopia to ensure BEmONC care.	Innovation fidelity
Shapiro et al. (37)	During the 2011–2012 academic year, 170 teachers of prekindergarten through second grade across all 15 elementary schools in a Pennsylvania school district (targeting approximately 4,000 elementary-school students) were trained in PATHS and asked to deliver it in accordance with the curriculum manual. Two grant-supported technical-assistance providers (TAs) were hired to support PATHS implementation. The TAs worked with teachers to schedule monthly (8 times during the school year) classroom observations (eight observations of each teacher during the school year). To assess fidelity the TAs completed the 19-item PATHS Monthly Implementation Rating Form (provided by PATHS developers) during each observation. For the purposes of this study, 10 of the items were used to examine overall lesson implementation quality [innovation fidelity], specific dimensions of implementation, and teacher characteristics.	Innovation fidelity
Hoekstra et al. (38)	Evidence-informed physical activity promotion program in Dutch rehabilitation care. Fidelity scores were calculated based on annual surveys filled in by involved professionals (*n* = ± 70). Fidelity scores of 17 organizations at three different time points. Three trajectories were identified as the following: “stable high fidelity” (*n* = 9), ‘moderate and improving fidelity’ (*n* = 6), and ‘unstable fidelity’ (*n* = 2).	Innovation fidelity
Chinman et al. (39)	Fidelity (adherence, quality of delivery, dosage) and the proximal outcomes of the youth participants (aged 10–14)—attitudes and intentions regarding cigarettes, alcohol, and marijuana use. Fidelity was assessed at all sites by observer ratings and attendance logs. Proximal outcomes were assessed *via* survey at baseline, 3, and 6 months. Fidelity was assessed at all sites by observer ratings and attendance logs. Proximal outcomes were assessed *via* survey at baseline, 3, and 6 months. A 2-year implementation support intervention. It compares 15 Boys and Girls Club sites implementing CHOICE (control group), a five-session evidence-based alcohol and drug prevention program, with 14 similar sites implementing CHOICE supported by GTO (intervention group).	Innovation fidelity
Rahman et al. (40)	In the first 3 months, functional water seals were detected in 33% (14/42) of latrines in the sanitation only arm; 35% (14/40) for the combined WSH arm; and 60% (34/57) for the combined WSH and Nutrition arm, all falling below the pre-set benchmark of 80%. Other fidelity indicators met the 65 to 80% uptake benchmarks. Rapid qualitative investigations determined that households concurrently used their own latrines with broken water seals in parallel with those provided by the trial. In consultation with the households, the authors closed pre-existing latrines without water seals, increased the CHWs’ visit frequency to encourage correct maintenance of latrines with water seals, and discouraged water-seal removal or breakage. At the sixth assessment, 86% of households in sanitation-only; 92% in the combined WSH; and 93% in the combined WSH and Nutrition arms had latrines with functional water seals.	Innovation fidelity
Fixsen and Blase (41)	Innovation fidelity for practitioners in 41 units assessed every six months for 10 years. Practitioners employed for 2 years or more remained at high fidelity each year even with turnover in staffing. Over 10 years, repeated measures noted substantial improvements for practitioners in the newly hired 0–6 month group and the 7-12 month group at each data point. The fidelity scores for these less experienced practitioners increased over 10 years from around 3 to over 5 on a 7-point scale.	Innovation fidelity; Sustainability
Jensen (42); Szulanski and Jensen (43)	Monthly performance data and two types of adaptation indicators, the addition of new routines and the modification of existing ones, for a majority of the U.S. units (approx. 3,500) of a large non-food franchisor. The results indicate that despite possibly increasing fit with the local environment, adapting recommended organizational routines results in poorer performance rather than greater. 5 data points in first year then every other year or so with 16 data points up to year 20.	Innovation fidelity; Sustainability
Althabe et al. (44)	Ten hospitals were assigned to the intervention group and nine to the control group. Pre-post assessments of changes in policies regarding active management of the third stage of labor, prophylactic use of oxytocin, or episiotomy as recommended by evidence-based obstetrical practices. The outcomes were measured at baseline, at the end of the 18-month intervention, and 12 months after the end of the intervention.	Innovation fidelity
McGovern et al. (45)	The Dual Diagnosis Capability in Addiction Treatment (DDCAT) index consists of 35 items, rated on a 5-point ordinal scale. Fidelity assessed using the DDCAT at three points in time over an 18-month period.	Innovation fidelity
Parvez et al. (46)	Enabling technologies and behavior change were promoted by trained local community health workers through periodic household visits. To monitor technology and behavioral uptake, the authors conducted surveys and spot checks in 30-35 households per intervention arm per month, over a 20-month period, and structured observations in 324 intervention and 108 control households, approximately 15 months after interventions commenced.	Innovation fidelity
Chaple and Sacks (47)	On average, programs (*n* = 150) received a follow-up assessment nearly 2 years after their baseline assessment. The baseline sample consisted of 603 outpatient programs licensed to operate in New York State. A follow-up sample of 150 programs was randomly selected to evaluate the impact of technical assistance and implementation support. Assessment Tools: The DDCAT6 and DDCMHT7 indices employed in this study included 35 items organized into 7 dimensions: (1) Program Structure, four items; (2) Program Milieu, two items; (3) Clinical Process—Assessment, seven items; (4) Clinical Process—Treatment, ten items; (5) Continuity of Care, five items; (6) Staffing, five items; and (7) Training, two items. Each item was scored on a 5-point scale of Dual Diagnosis Capability with three anchor points.	Innovation fidelity; Organization change
Aladjem and Borman (48)	Four evidence-based comprehensive school reform models designed for grades K–8 were studied in 170 “model” schools: Accelerated Schools (AS), Core Knowledge (CK), Direct Instruction (DI), and Success for All (SFA). Repeated measures over 5 years of teachers’ initial training, ongoing professional development, and external assistance from model developers/consultants. Assessment of the time allocated to an internal school staff member to facilitate and coordinate model implementation. The use of prescribed components of each model was assessed in each school at each time period.	Innovation Fidelity; Practitioner Training; Practitioner Coaching;
Forgatch and DeGarmo (49)	Evaluated innovation fidelity over the course of three generations of practitioners trained in PMTO. Generation 1 (G1) was trained by the PMTO developer/purveyors; Generation 2 (G2) was trained by selected G1 Norwegian trainers; and Generation 3 (G3) was trained by G1 and G2 trainers.	Innovation fidelity; Scaling
Kim et al. (50)	The authors used a cluster-randomized design with repeated cross-sectional surveys at baseline (2010, *n* = 2188), endline (2014, *n* = 2001), and follow-up (2016, *n* = 2400) in the same communities, among households with children 0–23.9 mo of age. Intervention exposure was measured by maternal recall of home visits (by BRAC frontline workers) received in the last 6 mo, number of times visited, attendance at a CM activity in the last 1 yr. and recall of ever having seen the A&T TV spots.	Innovation fidelity; Sustainability; Scaling
Litaker et al. (51)	In the setting of an intervention to increase preventive service delivery (PSD), the authors assessed practice capacity for change by rating motivation to change and instrumental ability to change on a one to four scale. After combining these ratings into a single score, random effects models tested its association with change in PSD rates from baseline to immediately after intervention completion and 12 months later.	Organization capacity
Parker (52)	Used established measures of job autonomy, skill utilization, participation in decision making, role overload to assess pre-post introduction of 3 lean production practices: lean teams, assembly lines, and workflow formalization. 4 groups surveyed over 3 yr period	Organization change
Jetten et al. (53)	Organization identification, Changing identity, Uncertainty, and Affect were assessed using an existing questionnaire. Administered about a month before and again about a month after a planned restructuring of work teams.	Organization change
Das et al. (54)	The facility assessment (readiness) tool focused on the infrastructure, training facilities, workforce, service delivery related to delivery and newborn care, practices, protocols, guidelines followed, communication, supplies, referral, and transport facility, documentation and reporting, and monitoring and supervision at the facilities. The authors used 26 maternal and newborn care signal function indicators, focusing on delivery, and postnatal care for assessing the readiness of the facilities for both routine and emergency care in health facilities. These 26 signal functions included general services and facilities (4 functions), routine obstetric care (3 functions), basic emergency obstetric care (5 functions), comprehensive obstetric care (2 functions), routine newborn care (3 functions), basic emergency newborn care (6 + 1 function), and comprehensive emergency newborn care (2 functions). Pre-post intervention assessment.	Organization change; Organization readiness
Ryan Jackson et al. (55)	Measuring implementation capacity at every level of the system for full and effective use of a practice that benefits all students is critical to alignment and cohesion of implementation efforts. Over 40 months, capacity is measured every 6 months using the State Capacity Assessment (SCA: Fixsen et al. (70)), Regional Capacity Assessment (RCA: St. Martin et al. (71)), District Capacity Assessment (DCA: Ward et al. (72)), and the school level, Drivers Best Practice Assessment (DBPA: Fixsen et al. (73)).	Organization change; system change; scaling; implementation teams; implementation capacity
Smith et al. (56)	Evaluate the comparative effectiveness of the Immediate vs. Delayed Enhanced REP sequences in 89 VA sites. Organization culture or climate measure at 6 and 12 months post-randomization. Organizational culture and climate measures came from the 2012 VA All Employee Survey (AES), a national survey of employees focused on organizational culture and climate distributed anonymously on a yearly basis.	Organization culture; Organization climate
Fixsen et al. (7, 22)	Assess the number of attempted replications of an evidence-based program over 10 years. Proximity discriminated early failures from successes (the group homes closer to the training staff got more personal, on-site observation and feedback). Given this, the focus shifted to developing Teaching-Family Sites instead of individual group homes. Longer term data showed that this had a large impact on sustainability (about 17% of the individual homes lasted 6 + years while over 80% of the group homes associated with a Teaching-Family Site lasted 6 + years).	Scaling; Sustainability
Massatti et al. (57)	IDARP is a longitudinal study with data gathered at approximately 9-month intervals. This analysis uses data gathered from the first three contact points. To collect information at each interval, a trained two-person team conducted confidential semi-structured interviews with multiple key informants. For organizations that discontinued their chosen evidence-based program, researchers asked key informants during the open-ended portion of the interview to provide the primary reasons for de-adoption.	Sustainability
Chilenski et al. (58)	PROSPER project intervention communities (*n* = 14) in Pennsylvania and Iowa composed the sample. Assessed TA collaboration (7 items); community readiness (15 items); Substance use norms (six items). 14 data points over 4.5 years.	TA Collaboration; Community readiness

## Discussion

It is heartening to see the breadth of implementation-specific variables that have been measured two or more times in one or more studies. Given the long-term complexities involved in providing implementation supports to achieve the full and effective use of innovations, repeated measurements of relevant variables are needed to promote a more complete understanding of implementation processes and outcomes. Yet, this exploratory review found few examples in the literature.

It is discouraging to see so few articles reporting repeated measures. The review found only 32 articles among the 3,950 mostly implementation evaluation articles, and provide an indicator of what must be done to advance the field. Implementation practice and science would be well served by investing in using and improving the measures identified in this review. The measures already have been developed and used in practice and appear to be relevant (they assess the presence and strength of an implementation-specific variable), sensitive (results showed change from one administration to the next), and practical (able to be administered repeatedly in practice). The field would benefit by using these measures in a variety of studies to establish more fully their consequential validity (does the variable being assessed impact the use and effectiveness of innovations). Meeting the criteria for action evaluations is a good start for the development of any measure.

As found in this study, there are good examples of repeated measures of implementation-specific variables in complex settings. Szulanski and Jensen (43) studied innovation fidelity and outcomes for over 3,500 franchises, each with 16 data points spanning 20 years for a total of 56,000 fidelity assessments that showed detrimental outcomes associated with lower fidelity in the early years and improved outcomes associated with lower fidelity after year 17. McIntosh et al. (35) studied innovation fidelity in 5,331 schools for 5 years, a total of 26,655 fidelity assessments that allowed the researchers to detect patterns in achieving and sustaining fidelity of the use of an evidence-based program. For 10 years Fixsen and Blase (41) studied innovation fidelity every six months for practitioners in 41 residential treatment units, a total of 820 fidelity assessments that detected positive trends among new hires as the implementation supports in the organization matured. Datta et al. (32) collected data for two years with 41 data points to track the effectiveness of continual attempts to produce improved outcomes for neonates admitted to the neonatal intensive care unit.

Innovation fidelity also has been assessed at an organization level. McGovern et al. (45) developed the Dual Diagnosis Capability in Addiction Treatment (DDCAT) to assess the dual diagnosis (substance abuse and mental health) capability of addiction treatment services. DDCAT items assess: (1) Program Structure; (2) Program Milieu; (3) Clinical Process: Assessment; (4) Clinical Process: Treatment; (5) Continuity of Care; (6) Staffing; and (7) Training. Organization dual diagnosis treatment capacity was measured at baseline and at 9-month follow-up in a cohort of 16 addiction treatment programs, 32 data points that found assessment, feedback, training, and implementation support were most effective in changing organization capacity. The DDCAT has been used in other studies by different authors to assess capacity (33, 47).

In these and other examples cited in this article, the measures of implementation variables are meaningful (relevant) and are repeated (practical) so that trends (sensitive) can be detected and corrected (if needed). Consequential validity was reported in these examples and in other articles (e.g., 43, 48, 49) and requires further study.

Innovation fidelity (*n* = 17) was the most frequent repeated measure. Innovation fidelity always is specific to the innovation under consideration. Implementation fidelity, on the other hand, refers to implementation-specific variables being used as intended. A science of implementation and assessments of implementation fidelity are intended to be universal. For example, the drivers best practices assessment (DBPA; 59, 60) measures the presence and strength of the implementation drivers (10, 15, 26, 27) that relate to (a) competency (selection, training, coaching, fidelity), (b) organization (facilitative administration, decision support data system, system intervention), and c) leadership (technical, adaptive). As shown in Table 2, over half of the measures (*n* = 30; Table 2) reported in the articles assessed one or more variables related to the implementation drivers. The DBPA has been used to assess implementation fidelity in a variety of settings and organizations, demonstrating a strong association with intended uses of evidence-based programs (61–64). As action measures are used in practice, the statistical (psychometric) properties can be assessed (61, 65).

These longitudinal studies are not typical, but they should be. After, before and after, one-time, or short-term assessments may be interesting but may add little to the science of implementation. To do something once or even a few times is interesting. To be able to do something repeatedly with useful outcomes and documented improvements over decades will produce socially significant benefits for whole populations (66). Data on the processes of implementation over time are badly needed.

There is much to be done to establish a science of implementation that has useful and reliable measures of implementation-specific independent (if…) and dependent (then…) variables. Implementation theory (67–69) can become the source of predictions (if…then) that can be tested in practice. In this way, like any science, a science of implementation can be cumulative and “crowdsourced” globally as theory-based predictions are tested and theory itself is improved or discarded.

## Limitations

In the current study, the AIRN EndNotes data base provided a convenient sample for the search that was conducted. Thus, the results of the search offer an indication regarding repeated measures of implementation variables. An exhaustive search of all available sources may produce a different view of the field.

## Data Availability

The original contributions presented in the study are included in the article/Supplementary Material, further inquiries can be directed to the corresponding author/s.
